# The role of *N*-glycosylation in B-cell biology and IgG activity. The aspects of autoimmunity and anti-inflammatory therapy

**DOI:** 10.3389/fimmu.2023.1188838

**Published:** 2023-07-27

**Authors:** Sara Trzos, Paweł Link-Lenczowski, Ewa Pocheć

**Affiliations:** ^1^ Department of Glycoconjugate Biochemistry, Institute of Zoology and Biomedical Research, Faculty of Biology, Jagiellonian University, Krakow, Poland; ^2^ Doctoral School of Exact and Natural Sciences, Faculty of Biology, Jagiellonian University, Krakow, Poland; ^3^ Department of Medical Physiology, Faculty of Health Sciences, Jagiellonian University Medical College, Krakow, Poland

**Keywords:** B cells, B-cell receptor (BCR), immunoglobulin class G (IgG), *N*-glycosylation, antibody-dependent cellular cytotoxicity (ADCC), complement-dependent cytotoxicity (CDC), autoimmunity, anti-inflammatory therapy

## Abstract

The immune system is strictly regulated by glycosylation through the addition of highly diverse and dynamically changing sugar structures (glycans) to the majority of immune cell receptors. Although knowledge in the field of glycoimmunology is still limited, numerous studies point to the key role of glycosylation in maintaining homeostasis, but also in reflecting its disruption. Changes in oligosaccharide patterns can lead to impairment of both innate and acquired immune responses, with important implications in the pathogenesis of diseases, including autoimmunity. B cells appear to be unique within the immune system, since they exhibit both innate and adaptive immune activity. B cell surface is rich in glycosylated proteins and lectins which recognise glycosylated ligands on other cells. Glycans are important in the development, selection, and maturation of B cells. Changes in sialylation and fucosylation of cell surface proteins affect B cell signal transduction through BCRs, CD22 inhibitory coreceptor and Siglec-G. Plasmocytes, as the final stage of B cell differentiation, produce and secrete immunoglobulins (Igs), of which IgGs are the most abundant *N*-glycosylated proteins in human serum with the conserved *N*-glycosylation site at Asn297. *N*-oligosaccharide composition of the IgG Fc region affects its secretion, structure, half-life and effector functions (ADCC, CDC). IgG *N*-glycosylation undergoes little change during homeostasis, and may gradually be modified with age and during ongoing inflammatory processes. Hyperactivated B lymphocytes secrete autoreactive antibodies responsible for the development of autoimmunity. The altered profile of IgG *N*-glycans contributes to disease progression and remission and is sensitive to the application of therapeutic substances and immunosuppressive agents. In this review, we focus on the role of *N*-glycans in B-cell biology and IgG activity, the rearrangement of IgG oligosaccharides in aging, autoimmunity and immunosuppressive therapy.

## 1 Introduction

B cells are an important component of immunity that links the innate and acquired immune response (1). B lymphocyte precursors are produced in the bone marrow and fetal liver, where they differentiate from hematopoietic stem cells (HSCs) to immature B cells (2–5). During the formation of transitional forms, pro-B and pre-B cells, the expression and rearrangement of the genes encoding V(D)J μ-heavy chain (μHC) and B-cell receptor (BCR) light chain occur (6). In addition, each cell is checked for proper receptor activity (positive selection) and for its inability to recognize self-antigens as non-self antigens (negative selection) (7, 8). This allows the BCR, which is the membrane-bound class M immunoglobulin (IgM), to recognize antigens it has never encountered before. Then, to complete differentiation, immature B cells with IgM surface expression, migrate to proliferation centers in peripheral lymphoid organs: spleen, lymph nodes, palatine tonsils and lymph nodules, where mature B lymphocytes expressing IgD and IgM are formed (6, 8, 9). The mature B lymphocytes upon contact with an antigen differentiate into memory B cells and plasma cells (plasmocytes) with the ability to secrete antibodies, which are essential for maintaining a defense response against antigens (10, 11).

However, a loss of immune tolerance to self-antigens, resulting in the development of autoimmunity, is increasingly observed. Hyper-reactive B cells present their antigens, secrete pro-inflammatory cytokines, and produce autoantibodies directed against specific organs and tissues (12). Most autoantibodies, responsible for the development of autoimmune diseases, are IgGs that are somatically mutated, suggesting that helper T cells (Th) drive the autoimmune B cell response (13).

Most proteins in the immune system, including crucial B cell molecules, such as Igs undergo glycosylation (14). Glycosylation is the most frequent protein post-translational modification (PTM) indirectly genetically mediated and dependent on age, sex, environmental and biochemical factors (15, 16). Basing on the type of bond between the protein backbone and the glycan, three types of glycosylation are distinguished: *N*-glycosylation, *O-*glycosylation, and *O*-GlcNAcylation, of which *N*-glycosylation is the most common (17, 18). *N*-glycosylation is characterized by the formation of an *N*-glycosidic bond between the *N*-acetylglucosamine (GlcNAc) of the glycan and the nitrogen of the asparagine (Asn) amide group within the Asn-X-Ser/Thr sequence, where X is any amino acid except proline. The monosaccharide donors are high-energy nucleoside diphosphates, mainly uridine diphosphate (UDP) and guanosine diphosphate (GDP) bound to sugar residues in the cytosol, except for sialic acid (SA) activated in the nucleus by cytidine monophosphate (CMP) (18–20).

**Figure 1:**
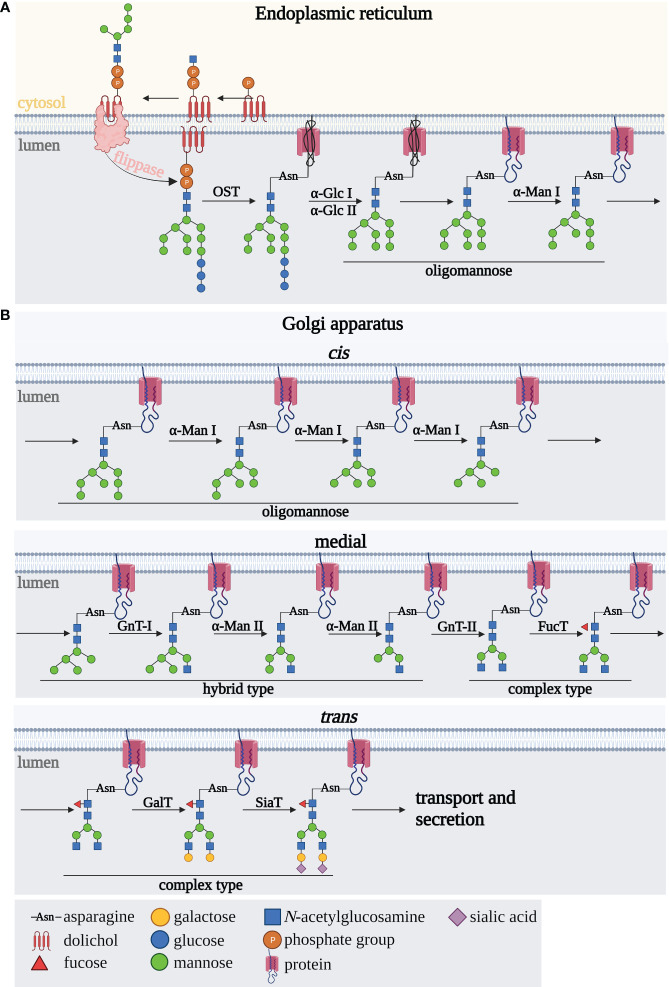
Scheme of *N*-glycan biosynthesis occurring in **(A)** the endoplasmic reticulum and **(B)** Golgi apparatus. A detailed description is provided in the text. αGlc I, alpha-glucosidase I; αGlc II, alpha-glucosidase II; αMan, alpha-mannosidase; FucT, fucosyltransferase; Gal-T, galactosyltransferase; GnT I, *N*-acetylglucosaminyltransferase I; GnT II, *N*-acetylglucosaminyltransferase II; OST, oligosaccharidyltransferase; SiaT, sialyltransferase.

*N*-oligosaccharide biosynthesis occurs in four main steps: synthesis of the oligosaccharide precursor, initiation of the polypeptide chain glycosylation, trimming of the oligosaccharide chain, and finally elongation leading to the formation of fully formed complex-type *N*-glycans. *N*-glycosylation pathway starts in the rough endoplasmic reticulum (rER) (**Figure 1A**) and continues in the Golgi apparatus (GA) (**Figure 1B**). A key role in the formation of the oligosaccharide precursor is played by the ER membrane-associated dolichol phosphate (P-Dol), to which, at an early stage of synthesis on ER cytoplasmic side, two GlcNAc residues are transferred to form GlcNAc2-P-Dol. The glycan formation is then followed by the attachment of five mannose (Man) residues due to the activation of mannosyltransferases (18, 19, 21) (**Figure 1A**). This oligosaccharide precursor is translocated into the ER lumen by flippases (22). A fully processed oligosaccharide precursor Glc3Man9GlcNAc2, formed after the attachment of four Man residues and three glucoses (Glc) inside the ER, is transferred to Asn of the *N*-glycosylation sequence within the polypeptide chain, via the enzyme complex of polypeptide oligosaccharyltransferase (OST) (21). In the subsequent steps involving glycosyltransferases and glycosidases, *N*-glycan remodeling occurs, first cotranslationally in the ER and then posttranslationally in the GA (21, 23). Glucose residues are detached enzymatically by glucosidases I and II giving an oligomannose oligosaccharide with nine mannoses, followed by Man removal by ER α1,2-mannosidase I (ERManI) and Golgi α1,2-mannosidases IA, IB, and IC, to form the Man5GlcNAc2 structure (**Figure 1B**). Then, it undergoes further remodeling by GlcNAc-transferase I, which attaches GlcNAc residues to the α1,3 arm of the glycan core. The formed hybrid-type *N*-glycan is then subjected to the action of mannosidase II, which removes two terminal Man residues, giving GlcNAcMan3GlcNAc2 structure in the medial GA. In subsequent steps, the outer part of the *N*-glycan is modified by the activity of GlcNAc-transferases, galactosyltransferases, fucosyltransferases and sialyltransferases with the various specificities to oligosaccharide acceptors and catalyzing the formation of the different glycosidic linkages. Depending on the expression and sequence of action of the glycosidases and glycosyltransferases in the subsequent steps of *N*-glycan biosynthesis, hybrid- and complex-type *N*-glycans are formed (18, 19, 21, 24, 25).

Despite the highly increased interest in the last decades, the analysis of B-cell protein glycosylation still is a rich field to explore. This review discusses the role of *N*-glycans in B-cell biology and function, including development, activation, and signaling. Furthermore, an impact of IgG glycosylation on its effector functions in antibody-dependent cellular cytotoxicity (ADCC) and complement-dependent cytotoxicity (CDC) is presented. Finally, the remodeling of *N*-oligosaccharide structures as the result of aging, autoimmunity, and treatment implementation in autoimmune diseases is discussed.

## 2 *N*-glycosylation regulates B-cell maturation and pre-BCR folding

**Figure 2:**
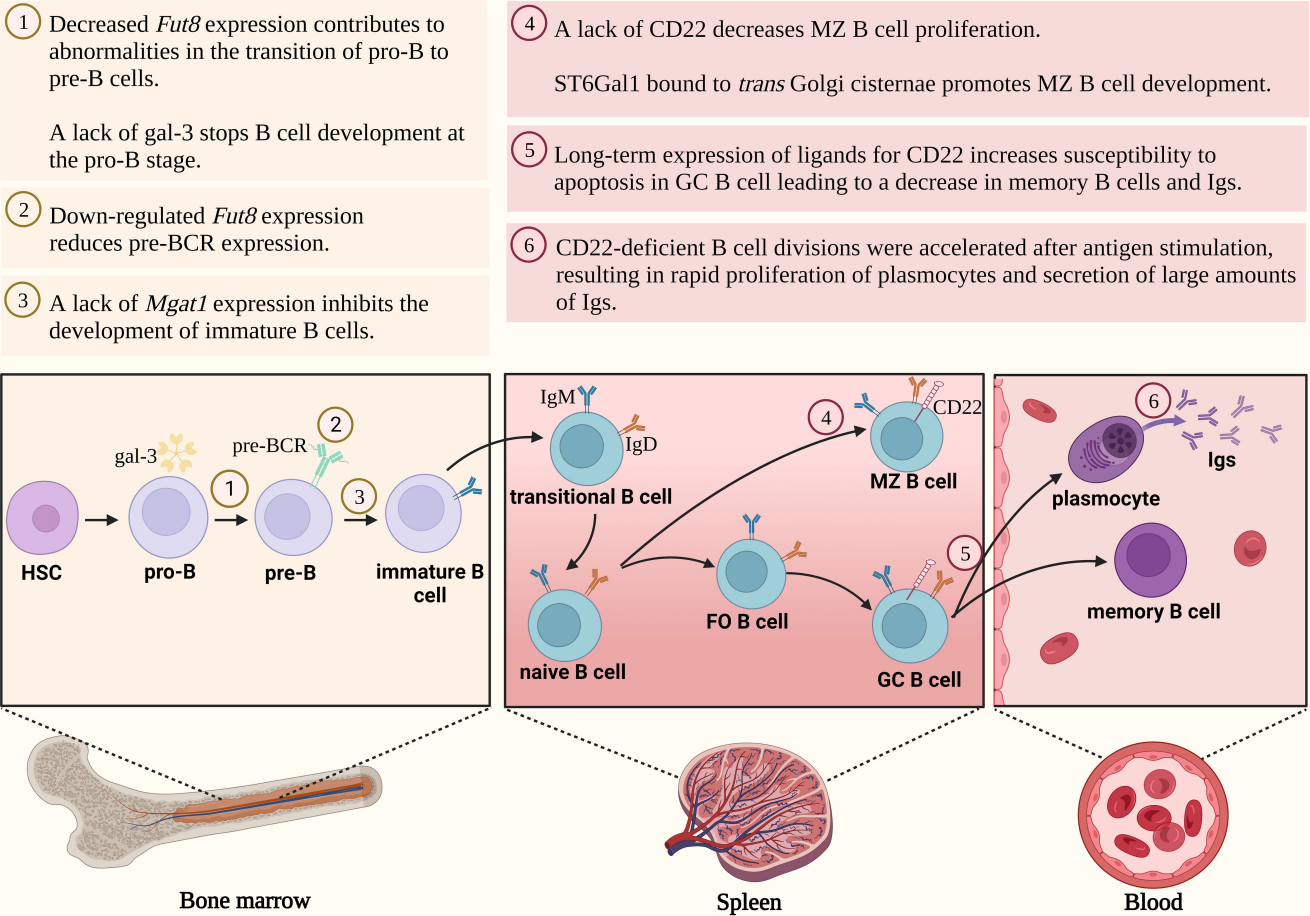
*N*-glycosylation regulates B cell development. FO B cell, follicular B cell; *Fut8*, gene encoding fucosyltransferase 8; GA, Golgi apparatus; gal-3, galectin-3; GC B cell, germinal center B cell; Ig, immunoglobulin; MZ B cell, marginal zone B cell; pre-BCR, pre-B cell receptor; *ST6Gal1*, gene encoding β-galactoside α2,6-sialyltransferase 1.

The first critical checkpoint in the early stages of B cell maturation is the differentiation of pro-B cells into pre-B cells (**Figure 2**). This process relies on the effective assembly and expression of the pre-B cell receptor (pre-BCR) on the surface of pre-B cells, which is based mainly on the rearrangements of the μ-heavy chain (μHC) gene) (26–28). The presence of pre-BCR is essential for the regulation of further B-cell proliferation and differentiation (29). Furthermore, a lack of pre-BCR expression leads to complete inhibition of pro-B cell maturation. The fully expressed pre-BCR complex is composed of μHC and the surrogate light chain (SLC), as well as Igα (CD79a) and Igβ (CD79b) signaling molecules (30–32). The μHC chain consists of a variable region (VH) and a constant domain (CH) (32). In contrast, the SLC comprises the non-covalently linked VpreB1/2 and λ5 fragments, which contain unique regions presented as arginine-rich tails protruding from the molecule (7, 32, 33). In the pre-BCR complex, the VpreB1/2 region binds to VH, while the λ5 domain interacts with CH1 μHC. Five potential *N*-glycosylation sites were recognized in the μHC chain, one of which is conserved at the position 46 (N46) (33). *N*-glycans identified in the μHC Ig chain are of the oligomannose type (29).

Mortales et al. showed that branched complex-type *N*-glycans are essential for the development of mature B cells in mice. Deletion of complex-type *N*-oligosaccharides in developing mice B lymphocytes by knock-out of *Mgat 1* gene encoding *N*-acetylglucosaminyltransferase I (GnT-I) (*Mgat1^f/f^/CD19-cre^+/-^* and *Mgat1^f/f^/CD23-cre^+^*), markedly reduced the percentage of cells in the bone marrow and spleen. Furthermore, the lack of *Mgat1* expression contributed to the inhibition of immature and transitional B cell development (34). Mice with a targeted disruption of the expression of the *Fut8* gene, encoding α1,6-fucosyltransferase 8 responsible for the attachment of fucose (Fuc) to the *N*-glycan core by α1,6-glycosidic linkage, showed abnormalities in the transition from pro-B cells to pre-B cells. Indeed, in *Fut8^-/-^* mice, bone marrow showed selective suppression in a subpopulation of pre-B cells without a concomitant change in pro-B lymphocytes (35). As appears, this is related to the effect of fucosylation on pre-BCR within the cell membrane (27). The expression of the pre-BCR complex was blocked in the *Fut8* knocked-out pre-B cells (70Z/3-KD cell line), and then restored upon reintroduction of the gene (70Z/3-KD-re cell line). In addition, the loss of α1,6-fucosylated *N*-glycans in the μHC chain reduced the interaction between μHC and λ5, as well as down-regulated the percentage of μHC-λ5-positive cells in the *Fut8^-/-^* pre-B cells (27). Fucosylation of the *N*-oligosaccharide core on the μHC of pre-BCR is also important for pairing with SLC. Pro-B cells from *Fut8*-deficient mice failed to fuse SLC to μHC to form pre-BCR. It is noteworthy, that not all pre-B cells produced in the bone marrow and fetal liver, are capable of producing pre-BCRs with a μHC chain paired with SLC (7). Therefore, poor μHC-SLC interactions result in low representativeness among the μHC Ig family (36). After μHC *N*-glycosylation, it is transported to the cell surface. In addition, the newly synthesised membrane form of μHC is resistant to endoglycosidase H (Endo H), while the secretory form is sensitive to this enzyme (37), which is responsible for the hydrolysis of the glycosidic bond between two GlcNAc residues in the core of the oligomannose *N*-glycans (38). Since developing B cells express different classes of BCRs that are composed of different HCs, Übelhart et al. demonstrated that the replacement of μHC by δHC led to a partial blockade of early B cell development caused by the inability of δHC to form a functional pre-BCR. Thus, the substitution of CH1 μHC in δHC enabled the formation of a functional pre-BCR complex. The conserved glycosylation site at position N46 in the CH1 domain of μHC is a regulatory element required for the formation of the pre-BCR receptor. Additionally, the N46Q mutant of the μHC chain was found to be fully functional, demonstrating that glycosylation of this conserved site is not required for the function of the pre-BCR complex (33).

Galectin (gal), a galactose-binding protein, also plays an important role in B-cell development (39). At an early stage of maturation, pre-B cells localise in niches with mesenchymal stromal cells secreting gal-1. This galectin is involved in synapse formation between pre-B cells and stromal cells by binding to the SLC of the pre-BCR receptor through direct protein-protein interaction and to stromal integrins through protein-glycan interactions. Thus, gal-1 serves as a ligand, which induces clustering of the pre-BCR receptor and promotes the progression of B-cell maturation (40, 41). However, the overall importance of gal-1 in B cell development *in vivo* remains somewhat unclear, as B cell development in the bone marrow was minimally impaired in *gal^-/-^* mice. Only when B cell development in the bone marrow of *gal^-/-^* mice was altered by the cytotoxic agent, hydroxyurea, a decrease in pre-B proliferation and differentiation was observed (31). Although β-galactosides are the main glyco-ligands for galectins, sialic acid linked to galactose (Gal) was shown to regulate its coupling by galectins (42). Alfa2,3-sialylation of the receptors expressed on pre-B cell surface strengthened their binding to gal-1 as shown by blocking of α2,3-SA by *Maackia amurensis* lectin (MAL II). In addition, the interaction of gal-1 with SA α2,3-bound glycans resulted in the dynamic conversion of galectin from an exogenous to an endogenous type lectin. Importantly, this selectivity switch can keep pre-B cells alive (39). A recent study focused on analyzing the *N*-glycan pool of human naive tonsil, germinal center (GC) and memory B cells using mass spectrometry and flow cytometry, based on labeling the cells with lectins that recognize specific sugar residues. All three B cell types were shown to express three- and four-antennary complex-type *N*-glycans with poly-*N*-acetyllactosamine (poly-LacNAc) structures, which can be recognized by multiple types of galectins. Indeed, the expression of poly-LacNAc by naive B cells and memory B cells corresponded to the strong binding of gal-1 and gal-9. While gal-1 also showed strong binding to GC cells, gal-9 demonstrated a marked reduction in this interaction (43). Gal-3 is also important in B cell maturation. *Gal-3^-/-^* mice have a higher proportion of pro-B cells that showed increased surface levels of interleukin-7 receptors (IL-7R). Therefore, it can be inferred that up-regulated IL-7R signaling caused by gal-3 loss leads to the arrest of B cell development in the pro-B stage (44).

To date, most research focused on the CD22 molecule and its effect on B cell maturation. CD22 (Siglec-2) belongs to the sialic acid-binding immunoglobulin-like lectin receptor (Siglec) and is expressed only on B cells (45–48). CD22 is a transmembrane glycoprotein composed of an extracellular domain consisting of seven immunoglobulin domains (D1-D7) with 12 *N*-glycosylation sites. The D1 domain (the most N-terminal) has a V-type Ig-like fold and recognizes α2,6-sialylated oligosaccharides (47). An intracellular part of CD22 is a tyrosine-rich domain (46, 48).

The CD22 molecule is poorly expressed on the surface of pre-B cells at the early stages of their maturation, however, the mechanisms responsible for its low level are still unclear. No changes in the number of mature B cells were observed in *CD22^-/-^* mice, but an increased susceptibility to apoptosis was demonstrated after anti-IgM stimulation *in vitro*. It is noteworthy that the increased B cell death after anti-IgM stimulation did not affect the immune response, as CD22-knockout mice produced adequate amounts of Ig after immunization with thymus-dependent antigen (49–51). However, the deficiency or absence of the CD22 molecule in certain cases can affect the dynamics of the thymus-dependent immune response. Onoder et al. showed that the divisions of CD22-deficient B cells were accelerated after antigen stimulation, which resulted in the rapid proliferation of plasmocytes and secretion of large amounts of Ig. Also, the formation of proliferation centers was faster during the initial phases of the immune response, but the number of plasma cells decreased over time (52).

Although no changes in the percentage of follicular B cells are observed in the lymph nodes and spleen, the bone marrow of CD22-deficient mice lacks recirculating B cells (49, 50, 53). Sinusoidal epithelium in the bone marrow expresses ligands for CD22 molecules. Masking CD22 ligands by administering CD22-Fc Ig to wild-type mice led to a reduction of mature B cells in the bone marrow showing that the CD22-ligand interactions are essential for maintaining the recirculating B cells population (54).

During maturation of B lymphocytes CD22 expression increases, with the highest level on the marginal zone (MZ) of B cell precursors located in the marginal sinus of the spleen (55, 56). A selective decrease in the percentage of MZ B cells was reported in CD22-knockout mice, which resulted in an attenuated immune response to thymus-independent antigens (49, 50, 57). CD22-deficient B cells also showed increased motility and chemotaxis toward selected chemokines. Altered chemokine reactivity or increased BCR signaling in cells deficient in the CD22 molecule may lead to impaired compartmentalization of MZ B cells (57). B cell glycoproteins are sialylated both intracellularly in the classical pathway via ER and GA and extracellularly in the bloodstream in a reaction catalyzed by the β-galactoside α2,6-sialyltranferase 1 (ST6Gal1). This sialyltransferase has various effects on B cell development; the membrane-bound form in *trans* Golgi cisternae is essential for MZ B-cell maturation, while its extracellular activity promotes transitional B cells of type 1 (T1) development in the bone marrow and spleen (58).

CD22 is also essential for the formation of B lymphocytes in GCs and the generation of memory B cells (59, 60). The microenvironment of GCs plays an important role in the development of antibody affinity. B cells in GCs have a unique pool of glycans compared to naive B cells and this glycan profile is rearranged during B cell development, especially in terms of *N*- and *O*-glycan sialylation. Macauley at al. analysed B lymphocyte binding to its ligands via CD22, during B cell maturation in GC. The study showed that the expression of the high-affinity CD22 ligand is selectively down-regulated only at GC B cell stage, and it increases in memory B cells (61). These results prompted Enterina et al. to study the functional role of CD22 ligand down-regulation in GCs. Using a mouse model that maintains CD22 ligand expression on GC B cells they showed that remodeling of glycan on CD22 ligands has a critical role in maintaining B cells in GCs. Long-term expression of ligands for CD22 induces higher GC B cell death, decreases the number of plasmocytes and memory B cells, and delays Ig affinity maturation, however, all of these processes depend on the sialylation of CD22 ligands. Mouse CD22 binds stronger to *N*-glycolylneuraminic acid (Neu5Gc) α2,6-linked to oligosaccharides than to α2,6-*N*-acetylneuraminic acid (Neu5Ac), which is converted to Neu5Gc by CMAH hydroxylase. Downregulation of CMAH in mice leads to Neu5Ac accumulation and results in the weaker CD22 interaction with sialylated glycoepitopes. Due to inactivation of *CMAH* gene in human, B cell binding via CD22 in GCs is differentially regulated than in mice, namely, it depends on the attenuation of sulfated glycans on B cells developing in GCs in contrast to naive B cells, which are abundant in this type of oligosaccharides (62).

## 3 *N*-glycosylation regulates signal transduction initiated by BCR

Once B cells reach maturity, the BCR receptor is expressed on their surface and is present in the form of isotypes - mainly IgM and IgD (11). Upon antigen recognition, BCR initiates the activation of B cells, which results in their proliferation and differentiation into antibody-producing plasmocytes (63). The BCR complex consists of two polypeptide chains of heavy (H) and light (L) membrane immunoglobulin (mIg) linked together by disulphide bridges (63–65). The H chain consists of a variable domain (VH) and constant domain (CH). In contrast, the L-chain consists of a variable region (VL) that includes the N-terminal segment and one constant domain (CL). The BCR has a crystallisation-capable fragment (Fc) consisting of CH2 and CH3, located in the C-terminal region, and an antigen-binding fragment (Fab) containing complementarity-determining regions (CDR) present in the N-terminal part. Fc is responsible for BCR effector functions, while Fab mediates antigen recognition (9, 66). The BCR complex also contains an Igα/Igβ heterodimer (CD79a/CD79b) (67). In each of the cytoplasmic tails of CD79a/CD79b an immunoreceptor tyrosine-based activation motif (ITAM) with two conserved tyrosines is present (65). The *N*-glycans identified in the BCR complex are of the complex-type (29), rich in α1,6-bound Fuc. Core fucosylation can affect the affinity and conformational changes of ligands to receptors, which would indicate that Fuc helps to control antigen recognition by BCRs (68–72).

**Figure 3:**
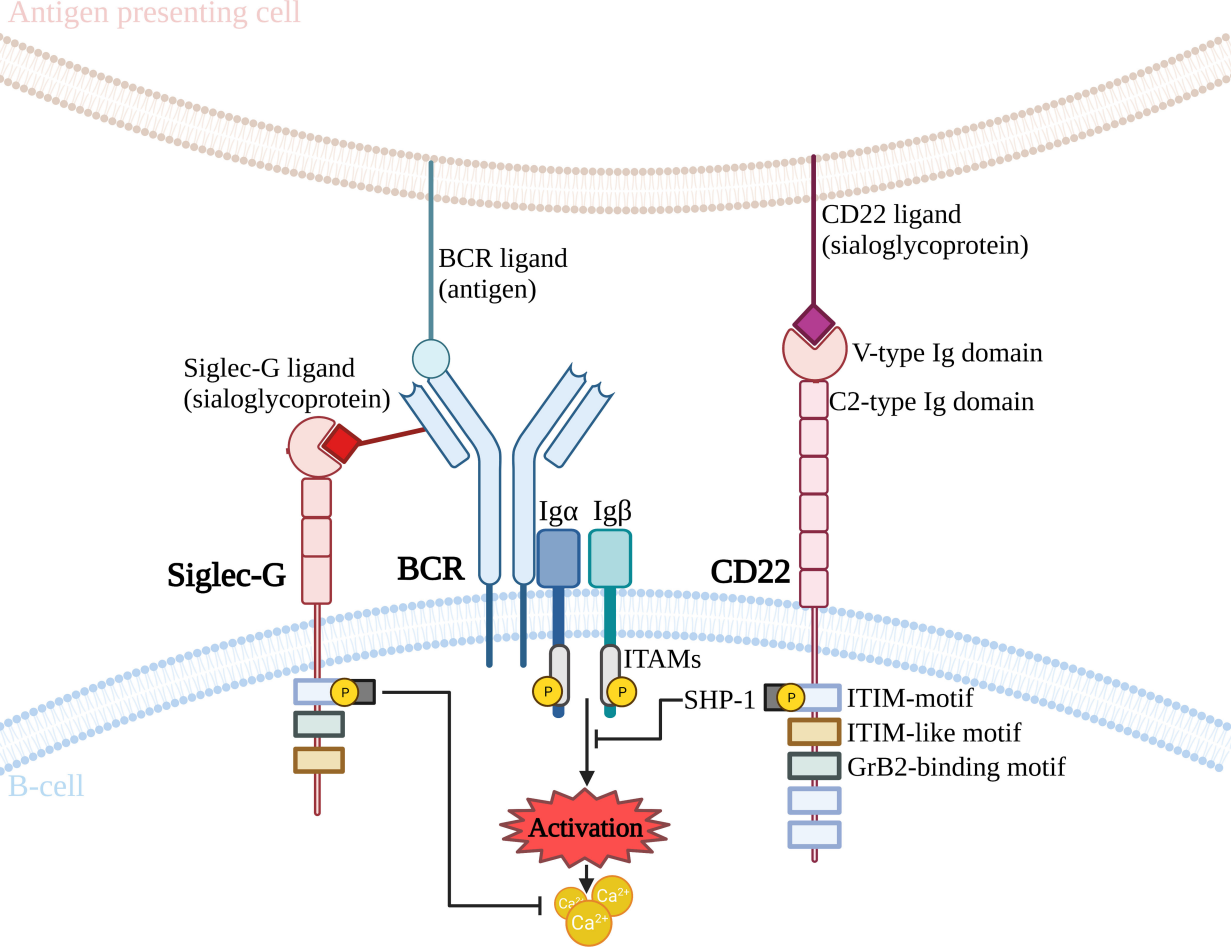
Effect of CD22 and Siglec-G interaction on BCR activation. BCR, B cell receptor; GrB2, Growth factor receptor-bound protein 2; Ig, immunoglobulin; ITAM, immunoreceptor tyrosine-based activation motif; ITIM, immunoreceptor tyrosine-based inhibitory motif; SHP-1, SRC-homology-2-domain-containing protein tyrosine phosphatase 1.

Another important significance of glycosylation has been reported for immunoregulatory glycoproteins which form a complex with the BCR. Prominent among these is the CD22 molecule (46, 48) (**Figure 3**). CD22 inhibits BCR receptor activity, due to its intracellular tyrosine-rich domain, which recruits the SHP1 (SRC-homology-2-domain-containing protein tyrosine phosphatase 1) phosphatase (73). Synthesis of ligands for CD22 requires the activity of a sialyltransferase, which catalyses the attachment of SA to the *N*-acetyllactosamine in *N*-glycan by an α2,6-bond (ST6Gal1) (74). Such a glycan ligand is found on many glycoproteins, among other present on CD22. Furthermore, it is quite common for CD22 molecules to bind to a glycan ligand present on neighboring CD22 (75). ST6Gal1 deficiency leads to the inhibition of BCR activity, resulting in suppression of the humoral type immune response. This is due to the lack of multimer formation between adjacent CD22 molecules, which promotes CD22 binding to the BCR. This configuration allows the SHP1 phosphatase to act and prevent the signaling from the BCR by phosphorylation of messenger proteins, thereby suppressing the immune response (74, 76, 77). It also contributes to the inhibition of autoimmune diseases and significantly improves survival in Lyn tyrosine kinase-deficient mice (77). Interestingly, the complete deletion of CD22 enhances BCR signaling and reverses the effect of ST6Gal1 loss, resulting in B-cell hyperactivation (45, 76). CD22 can bind glycan ligands in the *cis* (SA on the same cell surface), or *trans* (SA expressed on other cells) positions. The association of CD22 with a ligand in the *trans* position may modulate the folding of the glycoprotein complex and its localisation on the surface of B cells. In contrast, colocalisation of CD22 to the BCR receptor in the *cis* position probably occurs without the involvement of Siglec, which may involve protein-protein interactions mediated by the remaining extracellular domains of CD22 (56, 78). It is also worth noting the important function of the organization and dynamics of receptors expressed in the cell membrane, including the BCR, is signal transduction. Gasparrini et al., using super-resolution microscopy and single particle tracking, showed that CD22 cooperates with the cortical cytoskeleton in limiting BCR signaling. CD22 is found in highly mobile nanodomains that rapidly diffuse across the cell membrane. The formation of CD22 nanodomains was impaired due to reduced CD45 expression or was caused by mutation of the CD22 lectin domain, which is responsible for SA binding. The absence of the CD45 glycoprotein reduced the mobility of the nanodomains and increased the degree of CD22-CD22 association. Thus, CD22 cooperating with the cytoskeleton can rapidly control the BCR to balance activation or inhibition of signaling during naive B cell stimulation (79).

Siglec-G is another immunoregulatory glycoprotein that binds in complex to the BCR receptor (**Figure 3**). Mouse Siglec-G (a homolog of human Siglec-10), belonging to the CD33-bound Siglec family, is composed of an ITIM domain and an ITIM-like domain located in the cytoplasmic tail and additionally possesses a Grb2 binding site (80). Siglec-G is mainly expressed on B cells, where, like CD22, it negatively regulates BCR signaling (81–83). During the first studies in 2007 and 2014, mouse Siglec-G was described as a receptor that inhibits a subpopulation of B1 cells, but not B2 cells (81, 84). This has been demonstrated in Siglec-G-deficient mice, which show increased calcium signaling after BCR receptor stimulation only in B1 cells (81). Upon BCR receptor activation, the Siglec-G-IgM interaction is disrupted, revealing that glycan ligand binding is important for Siglec-G inhibitory functions. However, it is noteworthy that, unlike CD22, the loss of the ability to bind to a glycan ligand abolishes inhibition of B1 cell signaling and leads to a hyperactivated state (84). The limitation of this specific protein to bind to B1 cells is due to the fact that Siglec-G has a different binding pattern to SA than CD22, as it also recognises α2,3-linked SA (85). In addition, more α2,3-SA is expressed on the surface of B1 cells than on B2 cells (84). Loss of Siglec-G did not affect B2 cells, suggesting an inhibitory role for Siglec-G only on B1 cells (81).

## 4 Altered *N*-glycosylation of IgG in the light of its cytotoxic activity, the developement of autoimmunity and aging

### 4.1 IgG *N*-glycosylation

Plasmocytes represent the final stage of differentiation of mature B cells. They are most recognisable for their ability to produce and secrete antibodies – the major immune system glycoproteins (86, 87). The main class of human Igs is IgG, which is the most abundant *N*-glycosylated protein in blood serum and the most abundant class of antibodies in human plasma (88–90). The composition of the IgG *N*-glycans is influenced by both genetic and environmental factors, making it an excellent biomarker of overall human health. It seems likely that the process of IgG *N*-glycosylation in healthy individuals undergoes little change during homeostasis, while its disruption can be influenced by age, sex, sex hormones, pregnancy, menopause and stress. Moreover, changes in IgG *N*-oligosaccharide patterns have been implicated in disease progression and remission, representing both predisposition and functional mechanisms involved in the pathogenesis of diseases, including bacterial and viral infections, autoimmune diseases and cancers (91).

**Figure 4:**
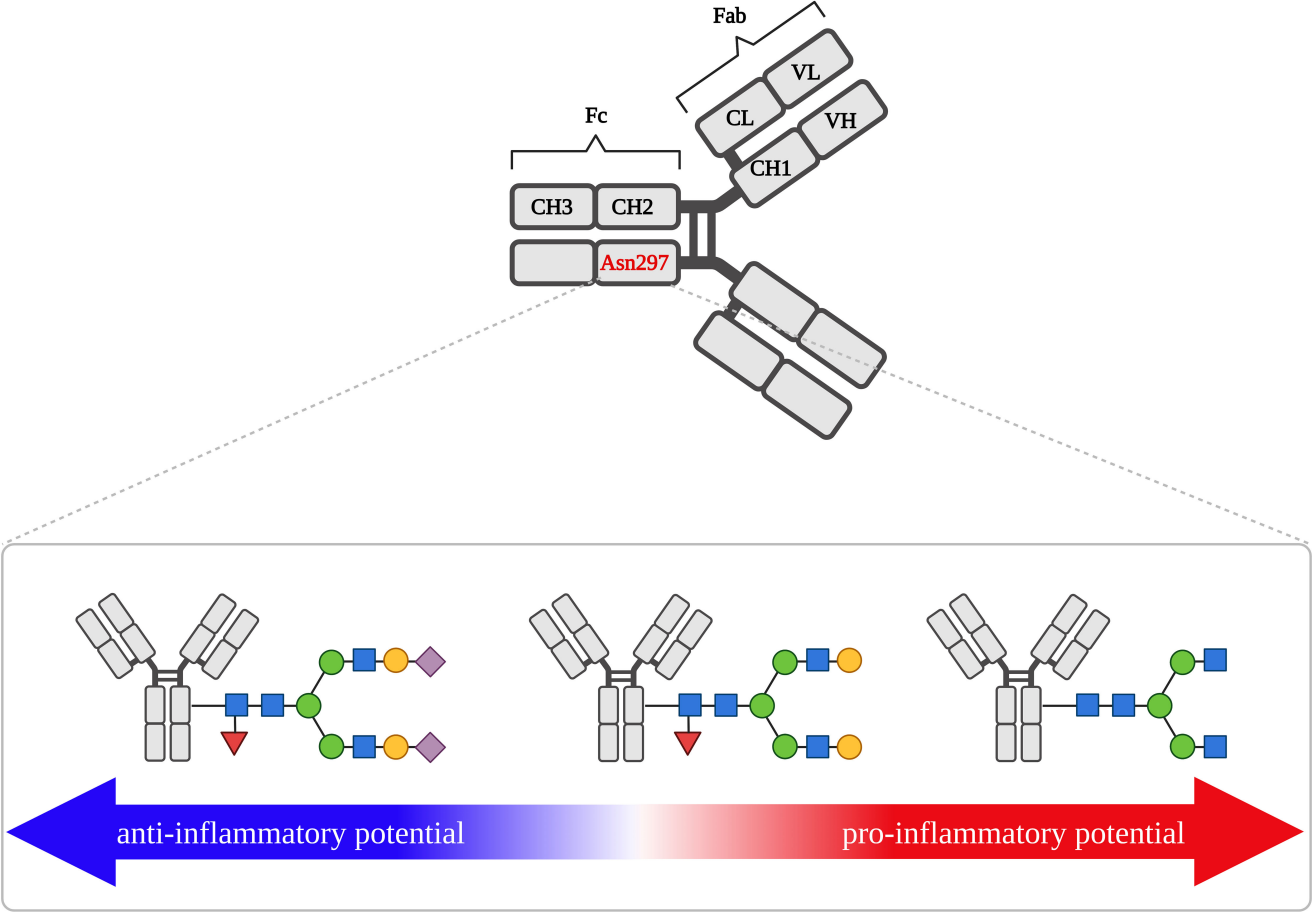
IgG structure and activity shift from anti- to pro-inflammatory, depending on the structure of *N*-glycan attached to Asn279 of Fc fragment. Asn279, asparagine 297; CH, constant heavy chain fragment; CL, constant heavy light fragment; Fab, antigen-binding fragment; Fc, crystallized fragment; VH, variable heavy chain fragment; VL, variable light chain fragment. Fucose (red triangle), galactose (yellow circle), mannose (green circle), *N*-acetylglucosamine (blue square), sialic acid (purple diamond).

Oligosaccharides are an integral part of the IgG molecule, constituting 15% of its molecular weight (91). IgG has a conserved *N*-glycosylation site located on Asn297 in the CH2 domain of the Fc fragment (**Figure 4**) (92). In approximately 30% of serum IgG *N*-glycosylation also occurs in the variable domain of the Fab fragment (93). Although not every antibody contains glycans in the Fab fragment, this post-translational modification is important in antigen binding by IgG (72). The presence of sugar residues in Fab usually increases the antibody affinity for the antigen, depending on the hypervariable site to which the oligosaccharides are attached. Sugar structures attached to Asn58 of the IgG HC variable region cause a 10-fold increase in affinity, glycans present at Asn60 increase affinity 3-fold, while the presence of oligosaccharides at Asn54 prevents IgG from binding to the antigen. *N*-glycosylation at the Fab fragment also prolongs IgG half-life in circulation (22, 72). In both the Fab and Fc fragments, mainly complex-type *N*-glycans are observed. The *N*-oligosaccharides of the Fab fragment have a higher content of bisected GlcNAc, higher levels of galactosylation and sialylation, and are less fucosylated than the Fc fragment (89, 94). This composition of *N*-glycan IgG results mainly from the greater accessibility of glycosyltransferases to the Fab *N*-glycosylation site compared to Fc, where Asn297 is hidden between the CH2 domains of the two CHs. In addition, the presence of oligomannose glycoforms has been identified in the Fab region (72).

IgG mediates effector function through its ability to interact with its Fcγ receptor (FcγR) expressed in effector immune cells and with the complement component 1q (C1q) complex (95). The interaction between IgG and FcγR receptors also plays an important role in inflammation and some autoimmune dysfunctions (96). An effect of IgG-FcγR binding is highly dependent on IgG Fc *N*-glycosylation, but also on FcγR glycosylation.

### 4.2 Structure and *N*-glycosylation of FcγR

FcγRs are transmembrane glycoproteins that belong to the immunoglobulin-like superfamily (97, 98). Activation or inhibition of the effector function of the Fc IgG depends on the FcγR subtype (99). FcγR receptors are classified based on signal transduction effect mediated through intracellular activation motif – ITAM (FcγRI, FcγRIIa, FcγRIIc, FcγRIIIa and FcγRIIIb) or the inhibitory motif – ITIM (FcγRIIb) (69, 97). Activating receptors are involved in the release of inflammatory mediators, ADCC, antibody-dependent cell phagocytosis (ADCP), and regulation of immune cell function (100–103). Inhibitory FcγR regulates the immune response by blocking the activation of basophils, mast cells, B cells, and monocytes induced by receptor triggering (104, 105).

FcγRs have at least two *N*-glycosylation sites depending on the type of receptor. FcgRIa contains *N*-glycans located at six Asn residues: Asn59, Asn78, Asn152, Asn159, Asn163, and Asn195. Despite the heavy glycosylation of FcγRIa, little is known about the exact structure of the oligosaccharides. However, as this receptor has a high affinity for IgG, it seems likely that *N*-glycans affect FcγRIa-IgG interactions (106, 107). FcγRIIa and FcγRIIc have two and three *N*-glycosylation sites, respectively (106, 108). FcγRIIIa and FcγRIIIb receptors contain *N*-glycan attached to Asn162. FcγRIIIa is stronger glycosylated which results from four additional *N*-glycosylation sites at Asn38, Asn45, Asn74, and Asn169. *N*-oligosaccharides bound to Asn45 and Asn162 of FcγRIIIa interact with Fc IgG and modulate its effector functions (106). *N*-glycan attached to Asn162 enhances FcγRIIIa-IgG binding through interaction with the glycans of the Fc fragment, while the *N*-oligosaccharide at Asn45 provides a spherical barrier to this interaction (106, 109, 110).

### 4.3 Importance of IgG *N*-glycosylation in ADCC

**Figure 5:**
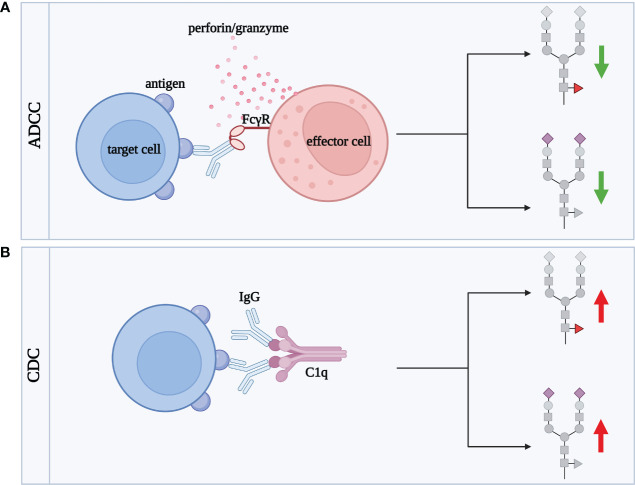
IgG sialylation and fucosylation influences the activation of ADCC and CDC cytotoxicity processes. Green arrows illustrate decrease of cytotoxic effect, red arrows correspond to increase of cytotoxicity. **(A)** ADCC involves effector cells, such as lymphocytes, macrophages, NK cells, eosinophils and dendritic cells, responsible for the lysis of target cells. Target cell death occurs as a result of the binding of the Fab fragment of IgG to a specific antigen present on the surface of the target cell. The IgG then binds via the Fc fragment to its FcγR receptor expressed in the membrane of the effector cell. This fusion allows the effector cell to contact the target cell and release cytotoxic granules, such as perforin and granzymes, which enter the target cell contributing to its death, **(B)** CDC is the process by which antibodies (mainly IgG1 and IgG3) lyse the target cell by activating a cascade of complement-related reactions. Activation occurs after binding of IgG Fab fragment to the antigen being expressed on the target cell surface. Subsequently, IgG via the Fc fragment binds to the complement component C1q, activating a cascade leading to target cell death. ADCC, antibody-dependent cellular cytotoxicity; C1q, complement component 1q; CDC, complement-dependent cytotoxicity; FcγR, receptor for the Fc fragment of antibodies; IgG, class G antibodies. Fucose, red triangle; sialic acid, purple diamond.

ADCC is the cytotoxicity process mediated by IgG, strongly dependent on *N*-glycosylation of the Fc fragment, which steers IgG activity into pro- or anti-inflammatory direction (111). Remodeling of IgG sugar structures affects ADCC by regulation of IgG binding to FcγRIIIa, which mediates pro-inflammatory response (**Figure 5A**) (112).

Fucosylation of Fc *N*-glycans plays a key role in IgG effector functions in the ADCC process. Core α1,6-linked Fuc attenuates the binding of the Fc fragment to FcγRIIIa and FcγRIIIb present on effector cells (113–115). In turn, afucosylated IgG shows up to 100-fold higher affinity for the FcγRIIIa receptor than core fucosylated glycoprotein (116, 117). The higher affinity of afucosylated IgG for the FcγRIIIa receptor leads to more intense cytotoxicity via the ADCC mechanism (93, 118).

Due to the significant impact of core fucosylation on IgG function, a manipulation of fucosylation became a useful tool to generate therapeutic monoclonal antibodies (mAbs) with the defined activity. One of them is Rituximab (RTX) directed against CD20 antigens expressed on B cells. It has been shown that the removal of the core Fuc from IgG1 *N*-oligosaccharide did not significantly affect binding to CD20, but enhanced the intensity of the ADCC response (119). IgG2 and IgG4 subclasses with deleted α1,6-Fuc acquired the ability to cell lysis via ADCC (119). A comparison of the fucosylated RTX glycoforms proved that antibodies with afucosylated complex-type *N*-oligosaccharides exerted the highest activity in ADCC, while those of the hybrid-type had the lowest cytotoxic potential. The differences in the intensity of the induced cytotoxic effect reflected the efficiency of RTX glycoform binding to the FcγRIIIa receptor (120). Afucosylation as a way to enhance the activity of therapeutic antibodies in ADCC was also tested for Pertuzumab, directed against the epidermal growth factor receptor (HER2), and applied in HER2-positive breast cancer therapy. Pertuzumab produced in a Chinese hamster ovary (CHO) cell line with *Fut8* knockout and subsequently desialylated enzymatically was used to test the impact of its sialylation and fucosylation on the cytotoxic activity. Sialylated Pertuzumab without core Fuc (Fuc^-^SA^+^) exerted a 6-fold higher cytotoxic effect than Fuc^+^SA^+^ glycovariant. The ADCC process was intensified up to 20-fold when Pertuzumab was non-fucosylated and desialylated (Fuc^-^SA^-^) in comparison to fully fucosylated and sialylated mAb, which proves that the presence of SA and Fuc favors anti-inflammatory potential of IgG (121).

Sialylation of IgG *N*-glycans is another oligosaccharide modification that profoundly affects the ADCC process (122). SA represents a small percentage of the sugar residues (10-15%) building *N*-oligosaccharides attached to the Fc fragment in the physiological state (91). Despite its low content, sialic acid also is an important player in the modification of ADCC intensity. SA attenuates ADCC due to the lower affinity of sialylated IgG for FcγRIIIa (88, 116, 123). Reduced IgG sialylation, accompanied by the higher pro-inflammatory activity of IgG, is characteristic of inflammation. The decreased binding of sialylated IgG to FcγR does not depend on the type of glycosidic bond (α2,3 or α2,6) which links SA to Gal (116). *In vitro* model of Hashimoto’s thyroiditis (HT), an autoimmune disease associated with inflammation in the thyroid gland, was designed to assess the effect of IgG sialylation on ADCC. Human thyroid epithelial cell line (Nthy-ori 3-1) was used as target cells, peripheral blood mononuclear cells (PBMCs) served as effector cells, and IgGs isolated from HT sera were an element of the model binding thyrocytes and immune effector cells. To investigate the effect of sialylation, IgGs were pretreated with neuraminidase (Neu), which removes SA. The results confirmed that desialylation of IgG *N*-glycans enhances the ADCC process, and showed that HT IgGs acted more effectively in inducing thyroid lysis than IgG from healthy donors (122), which probably resulted from their different sialylation and fucosylation profile described earlier (124). The inhibitory effect of desialylation on ADCC activity may result from the reduced IgG binding to the receptor due to the lower flexibility of the IgG hinge region (125). In turn, the results obtained for RTX did not show a significant effect of its modified sialylation on B-cell lysis via ADCC or on RTX affinity to FcγR, which reveals that studies on the effect of IgG glycosylation on ADCC are still needed, to clarify this issue (126).

### 4.4 Impact of IgG *N*-glycosylation on CDC

*N*-glycosylation affects IgG binding to C1q and activation of complement-dependent cytotoxicity (CDC) (**Figure 5B**). There is no doubt that both core fucosylation and sialylation of IgG significantly affect CDC, similar to ADCC described above. However, the results so far are inconsistent, and the contribution of individual monosaccharides of IgG *N*-glycans to this process is still unclear.

Natsume et al. using different isotypes of RTX in their study showed that an antibody variant consisting of the solid domain and hinge region of IgG1 and the Fc fragment of core fucosylated IgG3, was significantly more efficient in cell lysis via the CDC than non-glycoengineered IgG (127). The opposite results were obtained for a newly synthesised, afucosylated RTX (BLX-300), expressed in the Lemna aquatic plant-based system, which showed weaker cytotoxicity by CDC than RTX produced in the CHO cells (128).

Inconsistent results have also been obtained for IgG sialylation in CDC. One of the first studies showed that sialylated IgG1 has an increased affinity for the C1q complex (129). This was confirmed by the studies conducted in an *in vitro* CDC model with the use of HT IgG. The enzymatic removal of SA from IgG *N*-oligosaccharides significantly reduced thyrocyte lysis via the CDC (122). However, the results obtained for Pertuzumab revealed the opposite effect, namely the increased CDC after antibody desialylation (121). Furthermore, sialylation of the Fc fragment of IgG1 attenuated cytotoxicity through the complement system, suggesting that other sugar residues or other immune system factors are involved.

### 4.5 IgG *N*-glycosylation in aging

Changes of IgG glycosylation pattern are associated with aging, which has been supported by the fact that the glycome of this antibody reflects up to 64% of the variation in chronological age with an error of about 9.7 years (130). Galactosylation was the first and to date the most intensively studied IgG modification for which an age-related change in antibody activity has been reported. In one of the earliest studies, galactosylation level- was shown to increase from an early age, peak in early adulthood and then decline with advancing age (131, 132). However, inter-individual variability in IgG glycan composition is high, especially with regard to galactosylation (133). Results from studies on children samples are not consistent. This is mainly due to the fact that special approval from ethics committees is required to study biological material from children, making the number of samples for analysis insufficient to obtain reliable results. Several studies have reported an increased content of digalactosylated accompanied by decreased number of agalactosylated IgG sugar structures observed with age (131, 134). More detailed studies specified IgG4 as the only subclass which is affected by the changes in galactose content (135). In the case of studies on adult populations, it has been clearly shown that early adulthood is characterized by similar levels of IgG digalactosylation and agalactosylation (118). Importantly, a diverse IgG galactosylation was observed between genders for the adult population. Women start with galactosylation levels higher than men, which gradually reduce with age. After this period (after age of 40), the level of galactosylation continues to decline, but the total content of structures with Gal attached is higher in men than in women (130). Due to this finding, it was hypothesized that galactosylation level is related to hormonal status. This hypothesis was confirmed in a study that showed the estrogen replacement therapy implemented in women with menopausal symptoms prevented the reduction of IgG galactosylation (136).

Sialylation is the second IgG modification reported to undergo age-related alterations. Studies in pediatric populations have documented a down-regulation of glycan sialylation from the Fc region of IgG to the prepubertal period, after which SA content began to increase (118, 134, 135). However, a study that analyzed the entire pool of IgG sugar structures from the Fab and Fc regions found no age-related differences in IgG sialylation levels, indicating that the age-related change is driven by glycosylation of the Fc domain of IgG (136). Most studies on the adult population demonstrated a decrease in IgG sialylation after reaching early adulthood. Moreover, the dynamics in the change of gender-specific sialylation is similar to that observed for galactosylation (130, 137, 138).

Age-related changes of IgG fucosylation have also been studied, but the results are inconclusive. A study by Pučić et al. for subjects between 18 and 100 years of age showed no change in the total pool level of fucosylated core glycans with age (139). The results with the analysis for different age categories demonstrated that in childhood and adolescence, the amount of glycans with attached core fucose tended to decrease with age in both sexes. The level of core fucosylated oligosaccharides in the adult men population began to increase in early adulthood (20-30 years of age) and then decreased again. While in women, core fucosylation declined into early adulthood until around age of 40, when it began to increase. It was also found that women after age of 40 had lower levels of fucosylation than male peers, while around age of 60 the trend reversed (138).

Aging and age-related diseases are generally characterized by the increase of agalactosylated glycans, which are called pro-inflammatory IgG glycans. These pattern changes in IgG glycoforms that accompany aging are considered not only as one of the hallmarks but also as molecular drivers of the aging process. However, the regulation and mechanisms underlying age-related changes in IgG glycosylation and their role in aging remain not fully discovered.

### 4.6 IgG *N*-glycosylation is altered by severe autoimmunity

Over the past decade, it has been repeatedly shown that the glycosylation pattern of serum IgG varies significantly depending on the physiological state of individuals (95). The changes of IgG glycosylation have been reported in autoimmune diseases and chronic inflammatory conditions, with most of the results obtained for rheumatoid arthritis (RA) and systemic lupus erythematosus (SLE) (140–144).

As described above, the most significant impact on IgG activity exerts its core fucosylation and sialylation. Changes in IgG Fuc content during progression and remission of autoimmunity have been shown in SLE. Glycosylation analysis using *Aurelia aurantia* (AAL) and *Lens culinaris* (LCA) lectins in modified ELISA showed higher levels of the Fuc in IgG from SLE patients compared to healthy donors, and normalisation of IgG fucosylation in disease remission (145). Our study of immunoglobulin glycome in thyroid autoimmune diseases (AITD) by ultra-performance liquid chromatography-mass spectrometry (UPLC-MS) revealed the remodeling of IgG fucosylation in Hashimoto’s thyroiditis patients. HT-suffering patients with implemented L-thyroxine supplementation due to destructed gland tissue, showed down-regulation of two core fucosylated IgG oligosaccharide structures (F (6)A2G (4)2 and F (6)A2G (4)2S) and up-regulation of F (6)A2 *N*-glycan, compared to donors with elevated levels of thyroid autoantibodies but without changes in the structure of the thyroid gland (146).

Sialic acid is a negatively charged component of *N*-glycans that localizes terminally in their structure and is a crucial factor in protecting against autoimmunity. Therefore its reduction promotes proinflammatory IgG activity and in consequence the development of autoimmune disorders (147, 148). Lower SA content in IgG1 and IgG2 *N*-glycans was found in granulomatosis with vasculitis (GPA) (149). The level of sialylated *N*-oligosaccharides on the IgG molecule has a prognostic value in GPA relapse, as IgG1 sialylation decreased during disease progression and normalizes in remission (150). In sera of patients with inflammatory arthritis, the weaker IgG sialylation was also identified due to the decreased amount of sialylated triantennary *N*-glycans correlated with the reduction of inflammation (151).

Loss of sialic acid as a terminal monosaccharide is followed by agalactosylation of IgG in autoimmunity progression (152). Galactosylation of antibodies is another crucial modulator of their inflammatory activity. Agalactosylation of IgG in RA is the best-documented alteration of *N*-glycans in the autoimmune state, used as a prognostic parameter in monitoring disease development and progression. Individuals with lowered IgG galactosylation are more likely to develop RA. It has been estimated that dysregulation of the humoral immune response starts at least 3.5 years before the onset of the first RA-related symptoms and depends on IgG agalactosylation (91, 141). Agalactosylation of IgG was also described in HT progression in our study. Patients with Hashimoto’s thyroiditis showed a more abundant pool of agalactosylated *N*-glycans and reduced galactosylation on their IgGs, compared to donors with elevated levels of thyroid autoantibodies without symptoms of hypothyroidism (146).

### 4.7 IgG *N*-glycosylation is affected by immunosuppressive drugs

**Figure 6:**
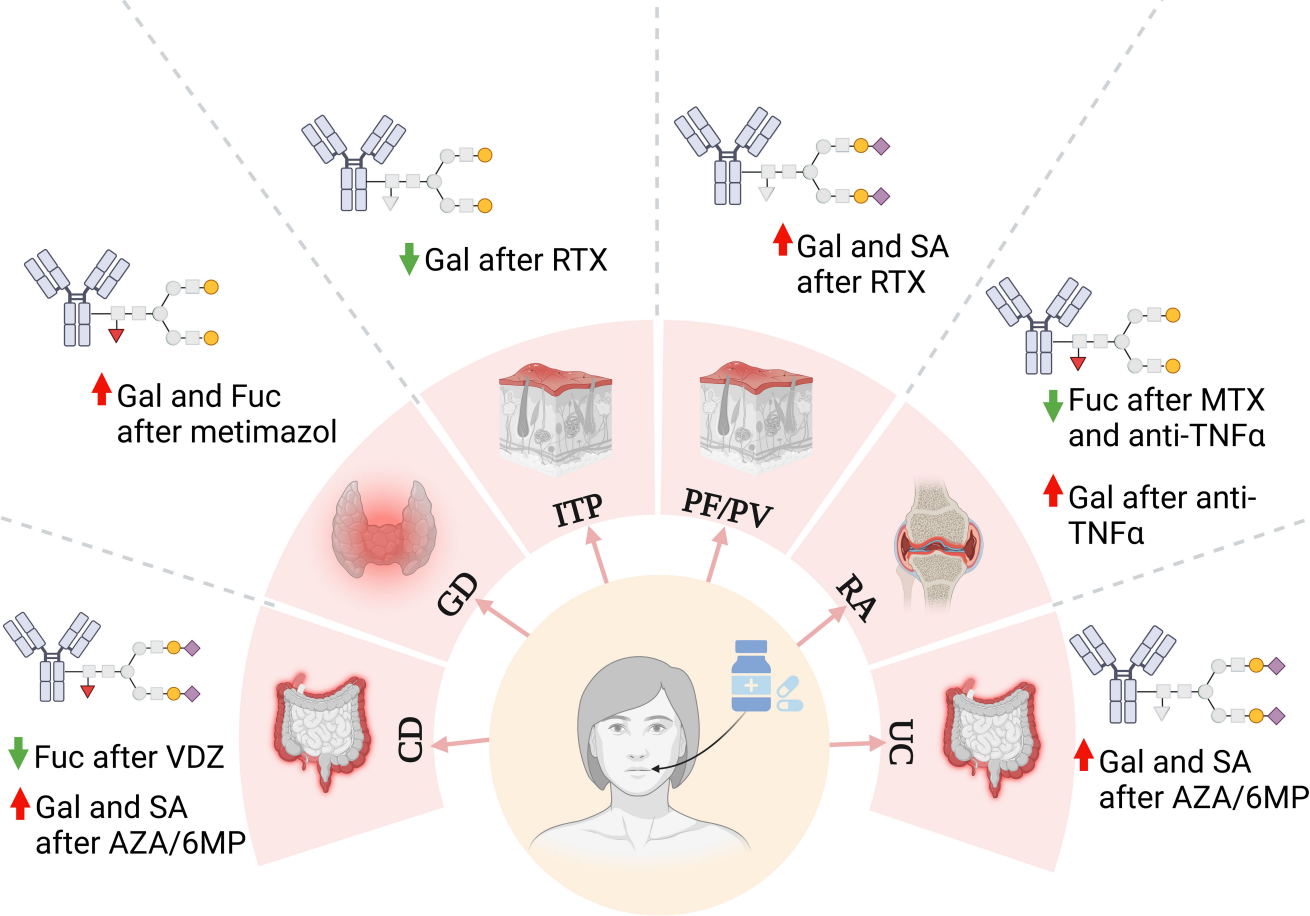
Changes in IgG *N*-glycan structures in autoimmune diseases after treatment implementation. AZA/6MP, azathioprine/6-mercaptopurine; CD, Crohn’s disease; Fuc, fucose (red triangle); Gal, galactose (yellow circle); GD, Graves’ disease; ITP, immune thrombocytopenia; MTX, methotrexate; PF, pemphigus foliaceus; PV, pemphigus vulgaris; RTX, rituximab; SA, sialic acid (purple diamond); TNFα, tumor necrosis factor α; UC, ulcerative colitis; VDZ, vedolizumab.

Changes in the IgG *N*-glycome occur not only during disease progression and remission but also during immunosuppressive treatment, where rearrangement of the sugar structures reverses the activity of the antibodies from pro- to anti-inflammatory (**Figure 6**) (142, 146, 151). One of the best-documented effects of immunosuppressive drugs on IgG glycosylation has been demonstrated for its fucosylation. Methotrexate (MTX) therapy routinely used in RA patients, decreased partially core fucosylation of IgG, which was up-regulated in disease progression as shown with AAL lectin in a modified ELISA (142). A similar effect of IgG fucosylation was observed in patients with arthritis treated with anti-TNFα (151). IgG fucosylation was also affected by vedolizumab (VDZ) – mAb directed against α4β7 integrin in Crohn’s disease (CD) therapy. The results showed a lower content of Fuc in IgG *N*-glycans, particularly in the FA2G2S2 structure purified from sera of patients after VDZ treatment compared to untreated patients (153). Alterations of IgG fucosylation were also observed in Graves’ disease (GD) patients as we documented by UPLC-MS analysis. Methimazole, a thyrostatic drug with immunosuppressive activity, increased the amount of core Fuc in GD patients after treatment compared to the same patients before drug administration, which probably contributed to IgG anti-inflammatory potential (146). Rituximab, mentioned above, is also used in the therapy of pemphigus, an autoimmune skin disease. And here, no significant differences in the IgG *N*-glycans fucosylation were observed between patients before and after 6- and 12-month RTX therapy (154).

As described in the previous sections, up-regulation of sialylation promotes the anti-inflammatory properties of antibodies. The results of some studies revealed that immunosuppressive therapy also affects IgG sialylation and redirects its activity to attenuate the immune response. A pool of disialylated IgG1 and IgG4 glycoforms was more abundant in patients with CD and ulcerative colitis (UC) treated with azathioprine/6-mercaptopurine (AZA/6MP), compared to those treated with anti-TNFα Ig. The different effects of treatment on IgG sialylation may result not only from the various mechanism of the applied drugs, but also from the different stages of disease progression when they were administrated, anti-TNF was assigned for those with severe disease, while AZA/6MP was applied for patients with milder symptoms (155). RTX in pemphigus therapy increased mono- and disialylated IgG structures compared to the patients before the implementation of the treatment (154). In the case of our research, methimazole did not affect the content of individual sialylated IgG *N*-glycans in GD, but the entire pool of mono- and disialylated *N*-glycans was significantly reduced in GD compared to healthy volunteers (146).

Impaired sialylation results from the lower IgG galactosylation, as sialyltransferases do not have acceptors to attach SA. Down-regulation of galactosylation and desialylation contribute to the pro-inflammatory potential of IgG, leading to more severe effects in autoimmunity (140). Drugs implemented in autoimmune diseases were also shown to restore IgG galactosylation. The results of our research showed well this reconstruction of IgG galactosylation in GD therapy. We identified more digalactosylated *N*-oligosaccharides accompanied by lower IgG agalactosylation as a result of methimazole treatment (146). Regalactosylation of IgG in immunosuppressive therapy was also reported in other autoimmune diseases, like RA and pemphigus (151, 154, 156). The increase of IgG galactosylation contributed to the attenuation of inflammatory processes in the patients treated with MTX and with a chimeric anti-TNFα antibody (142, 151, 156, 157). Enhanced IgG galactosylation has also been reported in patients with inflammatory bowel disease (IBD), CD and UC, treated with AZA/6MP compared to anti-TNFα Ig treatment (155), and in CD patients after VDZ implementation (153), while anti-TNFα therapy in CD resulted in the lower IgG agalactosylation (158). On the other hand, in immune thrombocytopenia galactosylation of IgG1 and IgG4 *N*-glycans in the blood of RTX-treated patients was reduced (159). Therefore, it is clear that immunosuppressive agents remodel IgG *N*-glycosylation, and the effect depends on the drug and the autoimmune disease.

### 4.8 Mechanisms of IgG glycosylation remodeling

It is well known that the pattern of IgG glycoforms changes throughout life, but the underlying mechanism(s) is(are) still unclear. Several hypotheses have been proposed to explain the increase in agalactosylation accompanied by the reduction of bigalactosylation and sialylation in IgG sugar structures during homeostatic imbalances.

The first hypothesis assumes that the structure of IgG glycans depends mainly on the expression of glycosidases and glycosyltransferases, and the availability of glycosylation sites for these enzymes in B lymphocytes and plasma cells responsible for antibody production (89). Of all the glycosyltransferases involved in the biosynthesis of IgG sugar structures, the contribution of β1,4-galactosyltransferase 1 (B4GalT1) to aging and the development of autoimmune diseases, have been most frequently observed (160–162). In addition, the relative reduction of antibody sialylation may result, at least to some extent, from the lower galactosylation, since SA attachment requires the presence of Gal in IgG *N*-glycans (163, 164). Some glycosyltransferases can form heteromeric complexes with each other in GA cisternae, among others B4GalT1 and ST6Gal1 were shown to form an enzyme complex to increase enzymatic activity (163, 164). However, to date, the expression of these glycosyltransferases in B cells has been studied to a limited extent, mainly because they make up a small percentage of peripheral blood cells (about 2%) in humans (165).

An important date in the history of IgG glycosylation research was 2016 year when it was established that IgG sialylation can be modified after the release of antibodies into the blood, independently of the classical intracellular process occurring in ER and GA of B and plasma cells. SA residues can be attached to IgG *N*-glycans as a result of extracellular post-translational modification occurring in the bloodstream with the participation of platelets. It was found that ST6Gal1, secreted into the blood by liver cells surrounding the central and portal veins, and cytidine monophosphate (CMP), the nucleotide donor of SA, which is released from platelet α granules, are involved in this process (166). However, a recent study by Oswald et al. challenged this theory, as animals lacking ST6Gal1 expression in liver cells and with undetectable ST6Gal1 in plasma retain normal α2,6-sialylation of IgG (167).

Following these first reports of IgG sialylation in the bloodstream, attempts have been made to investigate the activity of extracellular glycosyltransferases in aging and autoimmunity. To test the activity of the extracellular B4GalT1 and ST6Gal1 enzymes during aging, blood plasma from subjects ranging in age from 5 to 105 years was analysed. B4GalT1 activity was found to increase with age, while ST6Gal1 activity remained unchanged until around age 80, when it gradually began to increase. However, no correlation between the activity of these plasma enzymes and IgG glycan structure was shown, indicating that age-related differences in IgG galactosylation and sialylation are not directly related to the action of B4GalT1 and ST6Gal1 in circulation (168).

Our analysis showed that ST6Gal1 is present in sera from HT and GD patients and healthy donors and has the potential to modify IgG sialylation independently of the classical cellular glycosylation pathway (146). Changes in galactosyltransferase activity have also been studied in RA, which is the well-described autoimmune disorder with a higher content of agalactosylated glycans as the result of galactosylation decline. The results revealed a downward trend in the activity of galactosyltransferases in B cells compared to healthy individuals (169, 170). Modification of glycan structure by glycosyltransferases also depends on the presence of given sugar residues in the existing structure. Core fucosylation and antennae of IgG *N*-glycans enhance interactions between homologous domains of the Fc fragment mediated by sugar chains, which affects glycan conformation. This increases the stability of the Fc fragment facilitating access to the glycan by enzymes involved in downstream processing, including sialyltransferases. The efficiency of GlcNAc binding to *N*-glycan core was higher when it was preceded by core fucosylation (171). In contrast, an attachment of GlcNAc inhibits the activity of FUT8, which catalyzes the binding of core Fuc (172). Because glycosidases, responsible for removing monosaccharides from glycans, have also an effect on the final structure of IgG *N*-glycans (160), it was hypothesized that antibody agalactosylation could be attributed to increased activity of β-galactosidase, an enzyme that removes Gal residues. Analysis of β-galactosidase in plasma samples from healthy donors between the ages of 55 and 87 demonstrated that its activity increased with age, but it did not affect changes in IgG glycan structures (173). The possibility of IgG sialylation after release from plasma cells directly supports a model in which IgG glycans can be dynamically remodeled long after release into the circulatory environment. Indeed, it is now known that Neu1 and Neu3, neuraminidases responsible for removing SA from glycans, are also released into the circulation, although it is not yet clear whether these blood enzymes directly alters IgG sialylation (92).

The second hypothesis refers to the remodeling of IgG structures as a result of changes in the production or localization of activated nucleotide donors. However, despite available methods to quantify nucleotide donors, it has not been possible to confirm this hypothesis to date (161).

The third hypothesis assumes that changes in the glycosylation profile of antibodies result from the altered turnover of different IgG glycoforms mediated by neonatal Fc receptors (FcRn), asialoglycoprotein receptors (ASGPR), and mannose receptors (ManR) (174–176). FcRn is expressed in many human tissues, with the most abundant expression in vascular endothelial cells, where it extends the half-life of IgG in serum (177). Some studies indicated that similar to FcγR, FcRn’s affinity for binding IgG molecules is also modulated by IgG oligosaccharides (174, 175). Although FcRn expression appears to be modulated by both developmental stages and the immune context of the surrounding environment, no study to date has examined what happens to the rate of degradation of IgGs carrying various glycans during perturbations in organismal homeostasis (178).

Still none of the hypotheses put forward have been confirmed, so it cannot be said that any of them is closest to the truth. It also cannot be ruled out that all the proposed mechanisms do not interact with each other making up the final effect of IgG glycosylation. The involvement of some other mechanisms besides those mentioned above is also not excluded. Taking into account the importance of IgG glycosylation for its function, it is obvious that the mechanisms responsible for the rebuilding of antibody *N*-glycans will be intensively explored.

## 5 Conclusions

Glycobiological research has contributed significantly to defining lectin-glycan-based mechanisms responsible for the development and maturation of B cells. Indication of *N*-glycans as the main regulators of IgG activity was a milestone in glycoimmunology which redirected the research towards the analysis of *N*-glycan structure and function, not only in the humoral immune response, but generally in the immune system rich in glycosylated proteins. The number of data relating to B cell glycobiology is growing rapidly driven by new glycotechnologies, especially for IgG *N*-glycosylation significantly affected in inflammation and autoimmunity. We already know a lot about the glycobiology of B cell proteins, but for sure there is still more to be explored. A particular motivation to delve into this topic is the potential therapeutic applicability of basic research results through the development of glycoengineering-based therapies.

## Author contributions

ST wrote the first draft of manuscript and prepared the figures. EP and PL-L edited and reviewed the manuscript and figures. All authors contributed to the article and approved the submitted version.
